# Targeted ERAS implementation for postoperative care after Bellwether procedures in Africa: A pragmatic cluster-randomized trial from Ethiopia

**DOI:** 10.1101/2025.11.18.25339361

**Published:** 2025-11-19

**Authors:** Fitsum Kifle Belachew, Peniel Kenna Dula, Ermiyas Belay Woldesenbet, Betelehem Mulye, Desta Galcha, Kalkidan Kifle, Dagmawi Dagne, Megbar Dessalegn Mekonnen, Kokeb Desta Belihu, Tewodros Kifleyohannes, Brook Demissie, Abiy Dawit, Salome Maswime, Bruce Biccard

**Affiliations:** 1 Global Surgery Division, Department of Surgery, Faculty of Health Sciences, University of Cape Town, Cape Town, South Africa; 2 Global Partners for Improving Surgical System, Network for Perioperative and Critical Care (GPISS-N4PCc), Addis Ababa, Ethiopia.; 3 Department of Public Health, College of Medicine and Health Science, Wolkite, University, Wolkite, Ethiopia.; 4 Department of Clinical Epidemiology and Biostatistics, Faculty of Medicine Ramathibodi Hospital, Mahidol University, Bangkok, Thailand; 5 Quality Directorate, St. Peter Comprehensive Specialized Hospital, Addis Ababa, Ethiopia.; 6 Assistant Professor, Department of Surgery, Arba Minch University, South-Ethiopia.; 7 Department of General Surgery, ALERT Comprehensive Specialized Hospital, Addis Ababa, Ethiopia; 8 Department of Surgery, School of Medicine, Debre Markos Hospital Comprehensive Specialized, Debre Markos, Ethiopia.; 9 Network for Perioperative and Critical Care (N4PCc), Asrat Woldeyes Health Sciences Campus, Debre Birhan University, Debre Birhan, Ethiopia.; 10 Department of Anesthesia, School of Medicine, Asrat Woldeyes Health Science Campus, Debre Birhan University, Debre Birhan, Ethiopia.; 11 Department of Surgery, School of Medicine, Asrat Woldeyes Health Science Campus, Debre Birhan University, Debre Birhan, Ethiopia.; 12 Department of General Surgery, Department of Obstetrics and Gynecology, ALERT Comprehensive Specialized Hospital, Addis Ababa, Ethiopia; 13 Medical Service Hospital and Diagnostic Desk, Federal Ministry of Health, Addis Ababa, Ethiopia; 14 Department of Anaesthesia and Perioperative Medicine, Groote Schuur Hospital, University of Cape Town, Cape Town, Western Cape, South Africa.

**Keywords:** ERAS, Postoperative complications, Length of stay, Bellwether procedures

## Abstract

**Introduction:**

In Africa, where access to timely and safe surgical care remains limited, postoperative complications and prolonged hospital stays continue to challenge health systems. The Enhanced Recovery After Surgery (ERAS) protocol has been shown to improve perioperative outcomes by reducing hospital length of stay (LOS) and complications, but compliance remains inconsistent.

**Objective:**

To determine whether improving ERAS compliance in Ethiopia, through a “Triple Intervention Strategy” of early postoperative feeding, ambulation, and urinary catheter removal, could reduce hospital LOS for patients undergoing laparotomy and cesarean section (CS).

**Methods:**

This study was designed as a cluster-randomized clinical trial conducted across 10 hospitals within the National Perioperative Quality Improvement Network (NaPQIN) in Ethiopia. Hospitals were randomly assigned to either the intervention group (n=5), which received structured ERAS training reinforced through continuous monitoring and supervision, or the control group (n=5), which continued standard perioperative care without additional reinforcement. The primary outcome was hospital LOS, and secondary outcomes included compliance with the ERAS components, determinants of LOS, and postoperative complications. Data were managed through the NaPQIN platform and analyzed using R statistical software.

**Results:**

A total of 8,256 patients were enrolled, with 5,887 (71.3%) in the intervention group and 2,369 (28.7%) in the control group. Full compliance with the ERAS bundle improved to 76.5% in the intervention group compared to 57.9% in controls (p < 0.001). Patients in the intervention group had a significantly shorter LOS (mean 80.75 vs. 89.24 hours; p < 0.001). The intervention group also had significantly fewer postoperative complications (2.1% vs 4.8%; p < 0.001), and more patients were discharged without any complications.

**Conclusions and Relevance:**

This pragmatic trial, enabled by a national perioperative data system, demonstrated that the targeted implementation of postoperative ERAS elements, early oral feeding, mobilization, and timely urinary catheter removal significantly improved compliance and reduced hospital stay without requiring additional resources. While full ERAS pathways remain the ideal, focused, context-adapted strategies can offer scalable benefits in LMIC settings burdened by surgical backlogs and limited perioperative capacity. Broader adoption should prioritize tailored integration, ongoing evaluation, and provider engagement to maximize system-wide impact.

**Trial Registration:**

**pactr.samrc.ac.za** identifier PACTR202502863551536

## Background

Surgical care is a vital component of public health, as evidenced by the Lancet Commission on Global Surgery, Disease Control Priorities 3, and World Health Assembly resolution 68.15 (1). Between 2004 and 2012, major surgical procedures increased from 234.2 million to 312.9 million per annum (2). Despite the increase in surgical volume, adverse outcomes, including postoperative complications and mortality, remain significant concerns globally (3). Africa, home to many low- and middle-income countries (LMICs), has unique challenges in surgical healthcare, with opportunities to improve postoperative mortality and complications, currently reported at 2.1%, and 18.2%, respectively, despite lower surgical volumes and healthier patients than global comparators (4). Ethiopia exemplifies the challenge of delivering surgical care in a low-resource environment (5). The number of surgeries performed in Ethiopia’s public health facilities falls significantly short of the target of 5,000 surgeries per 100,000 population annually. This situation highlights the need for quality care initiatives such as the Enhanced Recovery After Surgery (ERAS) to enhance surgical care and improve patient outcomes (6).

ERAS is a multimodal approach to perioperative care that combines evidence-based practices, including preoperative counselling, minimally invasive surgical techniques, and early mobilisation, to optimise patient outcomes, reduce complications, and expedite recovery, thereby shortening hospital stays following surgery (7). Although research is limited, a systematic review and meta-analysis of gastrointestinal surgery suggests that ERAS practices in African settings offer similar benefits to those in high-income countries, although the dataset is small (8). These benefits include shorter hospital stays, reduced complications, and improved patient outcomes, which are crucial in resource-constrained settings to minimize surgery-related costs (8,9). A study of a perioperative registry in Ethiopia assessed compliance with ERAS protocols and found a 65.2% compliance rate for Bellwether surgeries (10). This suggests a significant opportunity to improve ERAS implementation in some LMICs to improve surgical outcomes.

In this trial, we aimed to determine whether improving ERAS compliance in Ethiopia through a bundled “Triple Intervention Strategy” of early postoperative feeding, ambulation, and urinary catheter removal for patients undergoing laparotomy and cesarean section would decrease hospital stay.

## Material and Methods:

This trial adheres to the 2010 Consolidated Standards of Reporting Trials (CONSORT) extension for cluster-randomized studies (11). The trial protocol is registered in the Pan-African Clinical Trials Registry (*PACTR202502863551536*) (12). Ethical approval was obtained from the University of Cape Town Faculty of Health Sciences Human Research Ethics Committee (*Protocol Number: 220/2024*) and the Arba Minch University Institutional Research Ethics Review Board Office (*Protocol Number: IRB/2320/2024*). Given that the study recommends strict compliance with the available guidelines and evaluates clinical practice patterns, the ethics committees waived the requirement for written informed consent. All data were de-identified and securely stored to ensure confidentiality.

### Trial design:

This study was a pragmatic, cluster-randomized clinical trial designed to evaluate the effectiveness of a structured training intervention on ERAS protocol compliance. The study was conducted across 10 hospitals participating in the National Perioperative Quality Improvement Network (NaPQIN), a collaborative platform supporting hospitals in implementing evidence-based perioperative practices, monitoring outcomes, and facilitating quality improvement initiatives (13). Hospitals were randomly assigned to either the intervention group (n = 5) or the control group (n = 5) using a computer-generated allocation sequence in R, conducted by an independent statistician. No stratification was applied in the randomization process.

The intervention group received structured ERAS training on implementing the intervention, as defined by the LMICs’ ERAS guidelines (14). Continuous reinforcement strategies included routine monitoring via the NaPQIN platform, supervision by designated site investigators, broadcast messages in recovery rooms and wards, and regular feedback and evaluation meetings. The control group received standard perioperative care without training or reinforcement interventions.

### Participants:

Eligibility included patients 18+ undergoing laparotomy or cesarean sections (CS) who consented. Exclusions were intraoperative deaths, ICU admissions or referrals post-surgery, procedures other than laparotomy or CS, or patients under 18. Recruitment from January to December 2024 involved tracking all registered patients’ surgical interventions and outcomes on the NaPQIN platform.

### Intervention:

Hospitals were randomized at the cluster level into either the intervention or control group using a computer-generated allocation sequence prior to the training. Following randomization, all participating hospitals in the intervention group received ERAS training as part of the NaPQIN initiative, ensuring a standardized approach to perioperative care.

### Training program

The ERAS training sessions, conducted by eight trainers who completed a ToT program, took place from December 6–9, 2023. The training included general surgeons, gynecologists, obstetricians, anesthesia professionals, and nurses from PACU, surgical wards, and gynecology departments. It emphasized safe OR practices and ERAS, with the Safe OR course developed to provide multidisciplinary training for safe surgery, uniting various perioperative healthcare providers who rarely train together (15). Launched in 2017, the course began as a three-day program aimed at improving surgical safety and team-based perioperative management. Although it briefly introduced ERAS principles, it did not fully cover their clinical implementation. For this study, the course was expanded to four days to include detailed ERAS protocols, NaPQIN platform, guidelines, recommendations, and strategies. It also retained core modules from the original Safe OR course on teamwork, safety, decision-making, patient optimization, anesthesia, analgesia, emergency management, recovery, and quality improvement. These sessions built a solid knowledge base for participating in hospitals.

### Reinforced implementation in the intervention group

Following training, the five hospitals assigned to the intervention group implemented a reinforced ERAS compliance strategy. To ensure structured compliance, lead hospital investigators were designated at these sites to oversee implementation and reinforce adherence.

The surgical team played a central role in leading protocol implementation, ensuring alignment between different perioperative teams, and promoting compliance with ERAS principles as part of routine patient care.

### Control group

The five hospitals assigned to the control group followed standard perioperative care practices without additional reinforcement strategies beyond the initial training.

### Outcomes

The primary outcome was hospital LOS, measured in hours from surgical admission until hospital discharge. Secondary outcomes included: i) compliance to ERAS protocol components, including early feeding, urinary catheter removal, and early ambulation, ii) factors influencing LOS, including patient demographics (age, ASA classification, urgency of surgery), surgical characteristics (procedure type, duration), and cluster-level variability, and iii) postoperative complications assessed using the Clavien-Dindo complication score system.

Data were collected from the NaPQIN platform, allowing for standardized, real-time data reporting across all study sites.

### Sample size

A *priori* sample size was determined using the Donner & Klar sample size formula to estimate the effect size for a continuous outcome (16). We proposed an ICC (intra-cluster correlation) of 0.02 based on the general recommendations due to the uncertainty of the correlation coefficient in epidemiologic studies (17). We determined that 1,000 patients (500 per arm) were required to detect a clinically meaningful difference in hospital length of stay, defined as a moderate standardized effect size of 0.4 (48-hour reduction in length of stay), with an alpha value of 5% and 80% power. Considering the estimated number of samples per cluster is 100, and assuming equal sizes across clusters, we enrolled 5 clusters per arm, totaling 500 patients per group and 1000 in total. We used the sample size determination formula coded in R(18). This function uses a “while loop” in R to adjust the number of clusters per arm iteratively, K_1_(set as zero as a starting point), until a stable value k is reached. The t-distribution was used instead of the Z-distribution. Although the minimum required sample size was 1,000, we included all eligible participants identified during the study period, resulting in a final sample of 8,256 patients.

### Blinding

Blinding was not feasible for investigators and healthcare providers due to the broadcast messages, reinforcement strategies, and direct involvement in ERAS compliance monitoring. However, statistical analyses were conducted with blinding, ensuring that the statistician remained unaware of group allocation to prevent bias in data interpretation.

### Statistical analysis

Descriptive and inferential statistical approaches were undertaken to assess factors influencing LOS and evaluate the differences between the control and intervention groups. Descriptive statistics were used to summarize baseline characteristics, with frequencies and percentages for categorical variables, while means (medians) with standard deviations (interquartile ranges) were used for continuous variables as appropriate. Group-level comparisons were conducted using the Chi-square test of independence, t-test, or ANOVA, as applicable, based on the type of variables compared.

The extent of missing data and its distribution were evaluated. Accordingly, we applied multiple imputations that considered the clustering effect on missingness by study clusters using Multiple Imputation with Chained Equations (MICE). After performing the imputation, the data generally preserves the pre-imputation data distribution in both categorical and numerical variables. To reduce the influence of extreme outliers, winsorization was made at the 10th and 90th percentiles prior to imputation. The normality assumption was checked using the Kolmogorov–Smirnov test.

For any deviation from the normality assumption, a non-parametric test was applied to compare the differences between groups.

To estimate the effect of intervention while adjusting for potential confounders, Generalized Estimating Equations (GEE) with an exchangeable correlation structure were used. This approach accounted for the clustering of patients within hospitals, adjusting for age, surgical type, educational level, urgency of surgery, and intravenous (IV) fluid volume. We have used a residual versus fitted values plot to assess model adequacy. Accordingly, a slight curvature was noted, indicating potential mild nonlinearity; however, the overall pattern suggests an acceptable model fit. Moreover, we have provided the log-transformed version of the model as a supplementary file.

In addition to individual patient-level analysis, cluster-level analyses were conducted to explore variability within and between hospitals. Ranked cluster means were used to provide further insight into group-level differences, highlighting institutional variations in ERAS adherence and LOS outcomes. All statistical analyses were performed using R statistical software version 4.4.2.

## Results

### Baseline characteristics

**Figure 1:**
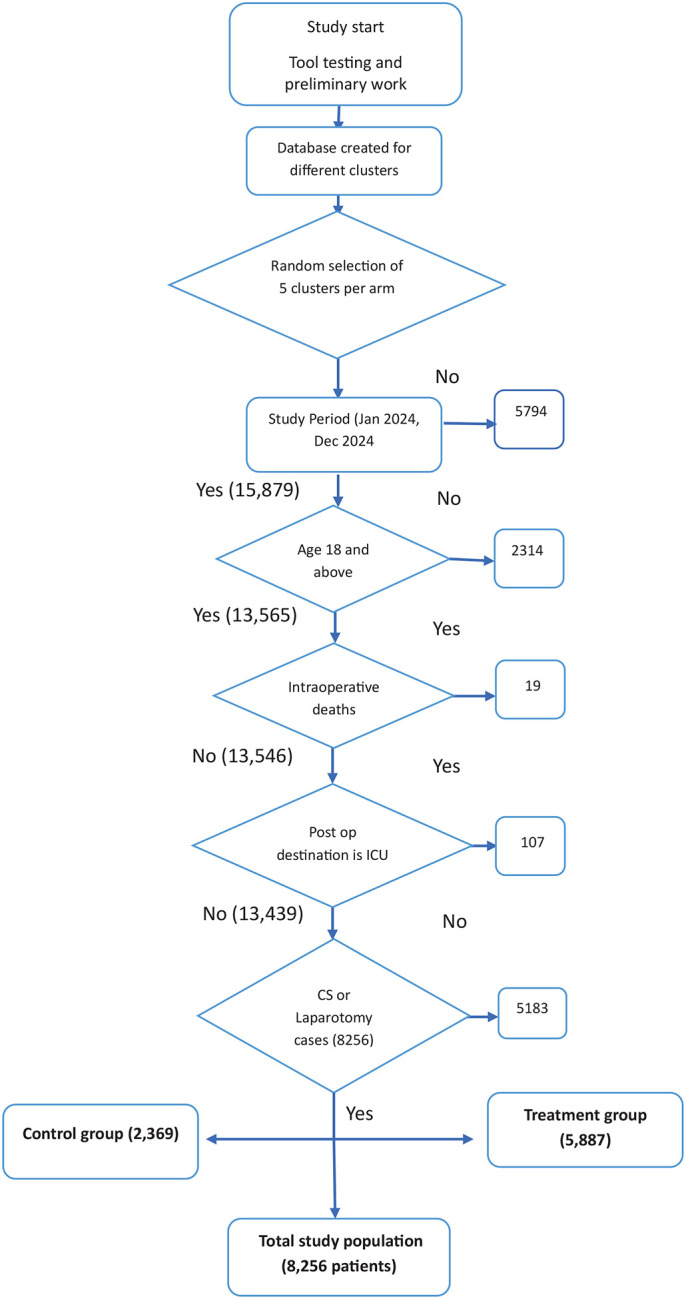
CONSORT diagram: screening, exclusion, and final study population

A total of 8,256 patients were recruited for the study from January to December 2024 (as shown in Figure 1), comprising 5,436 (65.8%) who underwent cesarean section (CS) and 2,810 (34.2%) who underwent laparotomy. The majority of patients were males, at 1,482 (52.7%). Of these, 5,887 (71.3%) were in the intervention group, while 2,369 (28.7%) were in the control group. There were differences between the two groups across all baseline characteristics (p < 0.001). Most procedures were non-scheduled, accounting for 6,759 (81.9%) of all surgeries, including 77.7% in the control group and 71% in the intervention group. All patients were classified as ASA I or II. Differences were observed across age, marital status, education, occupation, surgery urgency, and ASA class, as shown in Table 1.

Stratified comparisons by procedure showed no age or sex differences between intervention and control groups in CS and laparotomy cohorts (p > 0.05). Missing data across baseline variables was under 6% and was addressed with multiple imputations.

### Compliance with the ERAS interventions in practice

Full compliance with all three ERAS components was observed in 4,507 (76.5%) patients in the intervention group, compared to 997 (42.1%) in the control group (p < 0.001), as shown in Table 2. For compliance with each component, whether alone or combined, 5,196 (87.0%) of patients in the intervention group complied with early mobilization compared to 1,482 (61.0%) in the control group (p < 0.001). Early feeding was achieved in 5,231(96.2%) of the intervention group compared with 1,519 (64.2%) of the control group (p < 0.001). Compliance with catheter removal was 5,489 (93.2%) of the intervention group and 1,883 (79.5%) of the control group (p < 0.001).

### Hospital length of stay (LOS)

The overall hospital LOS was 83.19 (± 40.06) for patients in both groups. However, patients in the control group had a higher mean LOS of 89.24 hours compared to 80.75 hours in the intervention group (P<0.001), as shown in Table 3.

### Postoperative complications and patient outcomes

The intervention group had fewer postoperative complications than the control group (2.1% vs 4.8%; p < 0.001), mostly of which were minor (Clavien-Dindo grade 1). Although patients in the intervention arm had a higher number of grade II and III complications, the overall rate of postoperative complications was significantly lowered in the intervention group as shown in Table 4.

### Summary of factors influencing LOS

Table 5 summarizes factors associated with hospital length of stay. Being in the intervention group was significantly associated with a shorter stay (RR = 0.921, 95% CI: 0.887–0.949; p < 0.001). Longer surgery duration, undergoing laparotomy (RR = 1.30), older age, and higher volumes of IV fluids were all associated with an increased length of stay. In contrast, higher education levels and elective surgery (RR = 0.944, p = 0.001) were related to a reduced length of stay.

## Discussion

This trial evaluated a bundled ERAS approach with early feeding, mobilization, and catheter removal across 10 Ethiopian hospitals. It reduced hospital stays, with intervention patients discharged 8.49 hours earlier (p < 0.001). Compliance with all three increased from 42.1% to 76.5% (p < 0.001). Full compliance among CS patients shortened LOS by 7.6 hours, and early catheter removal and feeding among laparotomy patients reduced LOS by 6.6 and 13 hours, respectively.

These results show that ERAS principles can be successfully adapted to low-resource settings without requiring additional infrastructure or personnel. The bundled ERAS strategy significantly increased compliance, aligning with previous findings that partial ERAS implementation shortens recovery time (10). However, compliance was lower among laparotomy patients, particularly regarding early mobility, likely due to factors such as increased pain, surgical invasiveness, longer surgical duration, exhaustion, and urgency of surgery, issues consistent with other studies (18–20). This may also have been impacted by inconsistent team coordination and a lack of standardized reinforcement strategies, which have been shown to affect ERAS compliance in other contexts (21,22). A broader systematic review also highlighted the heterogeneity of ERAS implementation in LMICs, often driven by gaps in supervision, training, and local adaptation (23). In addition to reducing LOS, the intervention group also had significantly fewer postoperative complications, with most being minor. This further supports the clinical benefit of targeted ERAS compliance, particularly in improving postoperative recovery.

This study implemented most of the ERAS Society’s postoperative care elements for LMICs, excluding multimodal analgesia due to resource limits (14). It successfully integrated early oral feeding, mobilization, and urinary catheter removal, with real-time audit via Ethiopia’s registry, aligning with ERAS’s focus on feasibility and system monitoring (14). Lessons from the African Surgical Outcomes Study (ASOS-2) trial showed the challenges of implementing complex surveillance in resource-limited settings (24). The study demonstrated that a simplified ERAS bundle can be effectively integrated into routine care, offering measurable benefits. Although ERAS guidelines target elective procedures, findings suggest selected elements may also improve outcomes in non-elective surgeries, broadening their applicability.

The digital feedback loop reinforced behavior change, improved fidelity, and fostered local accountability, while also enhancing outcome measurement accuracy. The low ICC of 0.029 indicated minimal between-cluster variation, supporting generalizability across hospitals. Though the reduction in LoS was small, scaled benefits include better bed turnover, fewer surgical backlogs, and lower inpatient costs in overburdened LMIC hospitals (25,26). These findings support that perioperative pathways like ERAS can cost-effectively strengthen health systems, especially where resources are limited (27). Multivariable analysis showed the intervention (RR = 0.921), elective surgery (RR = 0.944), and higher education linked to shorter LOS, while older age, laparotomy, longer surgeries, and more IV fluids predicted longer stays. These build on prior research by identifying modifiable practices and patient factors affecting recovery (10).

The 8.5-hour LOS reduction, smaller than the 52-hour average in a recent LMIC meta-analysis (23), was achieved through a simple, low-cost protocol. While earlier studies in LMICs, including a trial in Uganda and an Ethiopian audit, reported larger LOS reductions (18 to over 120 hours) using more intensive or broader perioperative strategies, our results show that a simplified ERAS bundle can still yield system-level benefits when contextually adapted into routine care (10,21). This supports evidence from high-income settings where higher protocol compliance correlates with shorter LOS and better outcomes (28). With support from stakeholders, digital tracking can help LMICs contribute to global ERAS initiatives (29).

While the trial aimed to include all eligible surgical patients across the 10 participating hospitals, several real-world factors affected patient recruitment and group balance. High surgical volumes, limited data entry capacity, documentation gaps, and intermittent service disruptions, such as anesthesia stockouts, autoclave malfunctions, backup generator failures, hospital renovations, and climate-related or seasonal events, contributed to missed cases, surgical cancellations, and inconsistencies in patient recruitment. These challenges may have introduced selection bias, impacted data completeness, and limited the generalizability of the findings across all LMIC hospital settings.

This pragmatic, registry-integrated trial, one of the few cluster-randomized ERAS studies conducted in LMICs, demonstrated that reinforcing key postoperative elements can significantly improve compliance and shorten hospital stays, even in the absence of additional infrastructure or staffing. While comprehensive ERAS adoption remains the ideal, prioritizing high-impact elements provides a feasible starting point for African health systems facing surgical backlogs and limited perioperative capacity. Future efforts should focus on multidisciplinary, hospital-wide ERAS integration, supported by continuous data feedback, routine training, and aligned policy frameworks. Increased investment and collaboration will be essential to scale these strategies and assess their long-term clinical and health system impact.

## Supplementary Material

Supplement 1

- Appendix 1: ERAS Trial collaborators

## Figures and Tables

**Table 1:** Baseline characteristics of study participants; the intervention and control groups

	Control group, N = 2369				Intervention group, N = 5887			
Characteristic	N	CS N = 1,559^1^	Laparotomy N = 810^1^	p-value^2^	N	CS N = 3,877^1^	Laparotomy N = 2,010^1^	p-value^2^
Age group	2,369			<0.001	5,887			<0.001
21–30		1,152 (74%)	329 (41%)			2,969 (77%)	775 (39%)	
31–40		394 (25%)	172 (21%)			883 (23%)	462 (23%)	
41–50		13 (0.8%)	309 (38%)			25 (0.6%)	773 (38%)	
Sex	2,369			<0.001	5,887			<0.001
Male		0 (0%)	434 (54%)			0 (0%)	1,048 (52%)	
Female		1,559 (100%)	376 (46%)			3,877 (100%)	962 (48%)	
Marital status	2,369			<0.001	5,887			<0.001
Married		1,530 (98%)	584 (72%)			3,728 (96%)	1,501 (75%)	
Single		17 (1.1%)	208 (26%)			32 (0.8%)	392 (20%)	
Other		12 (0.8%)	18 (2.2%)			117 (3.0%)	117 (5.8%)	
Education level	2,369			<0.001	5,887			<0.001
Illiterate		138 (8.9%)	132 (16%)			571 (15%)	401 (20%)	
Elementary		254 (16%)	209 (26%)			903 (23%)	400 (20%)	
Highschool		578 (37%)	213 (26%)			1,060 (27%)	682 (34%)	
College and above		549 (35%)	235 (29%)			897 (23%)	241 (12%)	
Other		40 (2.6%)	21 (2.6%)			446 (12%)	286 (14%)	
Occupation	2,369			<0.001	5,887			<0.001
Government employed		205 (13%)	95 (12%)			410 (11%)	93 (4.6%)	
Private work		621 (40%)	178 (22%)			710 (18%)	576 (29%)	
Student		37 (2.4%)	92 (11%)			27 (0.7%)	176 (8.8%)	
Housewife		547 (35%)	173 (21%)			1,494 (39%)	314 (16%)	
Other		149 (9.6%)	272 (34%)			1,236 (32%)	851 (42%)	
Urgency of Surgery	2,369			<0.001	5,887			<0.001
Scheduled		311 (20%)	217 (27%)			874 (23%)	815 (41%)	
Non-scheduled		1,248 (80%)	593 (73%)			3,003 (77%)	1,195 (59%)	
ASA Class	2,369			<0.001	5,887			<0.001
I		249 (16%)	449 (55%)			1,175 (30%)	1,286 (64%)	
II		1,310 (84%)	361 (45%)			2,702 (70%)	724 (36%)	

Keys:

1 = n (%)

2= Pearson’s Chi-squared test

**ASA**- American Society of Anesthesiologist

**ERAS**- Enhanced Recovery After Surgery,

**NaPQIN**- National Perioperative Quality Improvement Network

**Table 2:** Adherence rate to the recommended ERAS triple elements

Treatment		Intervention group (N = 5887)	Control group (N = 2369)	Overall (N =8256)	P-value (Chi-square)
**Early Mobilization**	Yes	2774 (47.1%)	1483 (62.6%)	4257 (51.6%)	<0.001
	No	3113 (52.9%)	886 (37.4%)	3999 (48.4%)	
**Early Feeding**	Yes	3242(55.1%)	1509(63.7%)	4751(57.6%)	<0.001
	No	2645(44.9%)	860(36.3%)	3505(42.4%)	
**Catheter Removal**	Yes	4407(74.9%)	1843(77.8%)	6250(75.7%)	0.009
	No	1466(24.9%)	526(22.2%)	1992(24.1%)	
**Combined triple ERAS elements**	Yes	4507(76.5%)	1372(57.9%)	5879(57.9%)	<0.001
	No	1380(23.5%)	997(42.1%)	2377(28.8%)	

Keys:

ERAS- Enhanced Recovery After Surgery

N- Total population

**Table 3:** Summary of the study participants’ hospital length of stay (LOS)

Group	N	Mean	Median	SD	Min (OV)	Max (OV)	IQR	P-value
**Overall**	8,256	83.19	71.00	40.06	47.00	172.41	45.95	<**0.001**
**Control group**	2,369	89.24	72.08	44.02	47.00	172.41	63.27	
**Intervention group**	5,887	80.75	70.00	38.08	47.00	172.41	44.13	
**Control vs intervention (T-test)**								

Keys:

IQR- Interquartile range

Max - Maximum

Min -Minimum,

N - Number of participants,

OV- Observed values

SD - Standard Deviation,

**Table 4:** Postoperative complications by Clavien-Dindo classification across study groups

		Control group, N = 2369	Intervention group, N = 5887	p-value
No complication		2,255 (95.2%)	5,763 (97.9%)	<0.001
With Complication	1	87 (3.7%)	47 (0.8%)	
	2	19 (0.8%)	60 (1.0%)	
	3	8 (0.3%)	17 (0.3%)	

**Table 5:** Factors influencing hospital length of stay description of intervention and control groups

Term	Coefficient	Std. error	RR	P value	95% CI
**Intervention group**	−0.082	0.016	0.921	<0.001	(−0.113, −0.051)
Duration of surgery in minutes	0.003	0.000	1.003	<0.001	(0.002, 0.004)
Surgery type - Laparotomy	0.263	0.030	1.300	<0.001	(0.205, 0.321)
Education level - elementary	−0.038	0.015	0.963	0.011	(−0.067, −0.009)
Education level - high School	−0.078	0.015	0.925	<0.001	(−0.107, −0.048)
Education level - college and above	−0.075	0.015	0.928	<0.001	(−0.105, −0.045)
Education level other	−0.121	0.026	0.886	<0.001	(−0.172, −0.071)
Age category (31–40 years)	0.035	0.011	1.036	0.002	(0.013, 0.057)
Age category (41–50 years)	0.118	0.018	1.126	<0.001	(0.083, 0.154)
Total IV fluid (≤ 1000 ml)	0.047	0.018	1.048	0.009	(0.012, 0.082)
Total IV fluid (1000–1500 ml)	0.059	0.021	1.061	0.006	(0.017, 0.101)
Total IV fluid (1500–2000 ml)	0.089	0.020	1.093	<0.001	(0.050, 0.128)
Urgency of surgery - non-elective	−0.057	0.018	0.944	0.001	(−0.092, −0.023)

Keys:

CI - Confidence Interval,

RR – Risk Ratio,

Std. error - Standard error
